# Пролактолиберин увеличивает тревожность крыс

**DOI:** 10.14341/probl12770

**Published:** 2021-09-19

**Authors:** Д. А. Жуков, А. Г. Марков, Е. П. Виноградова

**Affiliations:** Институт физиологии им. И.П. Павлова РАН; Биологический факультет СПбГУ; Биологический факультет СПбГУ

**Keywords:** пролактолиберин, интраназальное введение, тревожность, социальное предпочтение, крыса

## Abstract

**ОБОСНОВАНИЕ:**

ОБОСНОВАНИЕ. Пролактолиберин, или пролактин-рилизинг-гормон (Прл-РГ), помимо стимуляции выработки пролактина, взаимодействует с различными отделами ЦНС, участвуя в реализации многих функций, отражающихся в поведении.

**ЦЕЛЬ:**

ЦЕЛЬ. Поскольку в литературе нет данных о связи Прл-РГ с тревожностью, целью данной работы явилось изучение влияния Прл-РГ на тревожность белых крыс Вистар.

**МАТЕРИАЛЫ И МЕТОДЫ:**

МАТЕРИАЛЫ И МЕТОДЫ. Тревожность оценивали в двух тестах. В приподнятом крестообразном лабиринте регистрировали время, проводимое в открытых рукавах и количество реакций свешивания, В тесте социального предпочтения регистрировали время, проводимое возле чужака, возле знакомой особи и на нейтральной территории.

**РЕЗУЛЬТАТЫ:**

РЕЗУЛЬТАТЫ. Введение Прл-РГ в дозе 10-10 М объемом 10 мкл в каждую ноздрю уменьшало время, проводимое животными в открытых рукавах приподнятого крестообразного лабиринта, и количество реакций свешивания. Для тестирования социального взаимодействия животных предварительно отбирали по высокому либо низкому уровням тревожности в тесте с приподнятым крестообразным лабиринтом. У крыс с изначально низким уровнем тревожности Прл-РГ уменьшал время, проводимое рядом с незнакомцем, что указывает на повышение уровня тревожности. Поведение крыс с изначально высоким уровнем тревоги не менялось после введения Прл-РГ.

**ЗАКЛЮЧЕНИЕ:**

ЗАКЛЮЧЕНИЕ. Результаты наших экспериментов свидетельствуют о том, что интраназальное введение Прл-РГ увеличивает тревожность крыс.

## Abstract (en)

**BACKGROUND:**

BACKGROUND: Prolactin-releasing peptide(Prl-RP), in addition to stimulating the production of prolactin, interacts with various parts of the central nervous system, participating in the implementation of many functions that are reflected in behavior.

**AIM:**

AIM: The effect of Prl-RP on the anxiety of white Wistar rats was studied since there were no data in the literature on the relationship between Prl-RP and anxiety.

**MATERIALS AND METHODS:**

MATERIALS AND METHODS: Anxiety was assessed in two tests. In the elevated plus-maze (EPM), the time spent in the open arms and the number of edge reactions were recorded. In the social preference test, the time spent near a stranger, near a familiar individual, and in neutral territory were recorded.

**RESULTS:**

RESULTS: The administration of Prl-RP at a dose of 10-10 M with a volume of 10 µl in each nostril reduced the time spent by the animals in the open arms of the EPM, and the number of edge reactions. For testing the social interaction, animals were pre-selected for high or low levels of anxiety in the EPM. In rats with initially low levels of anxiety, Prl-RP reduced the time spent near a stranger, indicating an increase in anxiety levels. The behavior of rats with initially high levels of anxiety did not change after application of the Prl-RP.

**CONCLUSION:**

CONCLUSION: The results of our experiments indicate that the intranasal administration of Prl-RP increases the anxiety of rats.

## ОБОСНОВАНИЕ

Пролактолиберин (Прл-РГ) — гипоталамический нейрогормон, выделенный из экстракта гипоталамуса крупного рогатого скота [1][2], увеличивал синтез пролактина в клеточной линии, полученной от аденомы гипофиза крыс, и в клетках гипофиза лактирующих крыс [3]. Кроме того, инъекция Прл-РГ стимулировала уровень пролактина в плазме крови у самок крыс в проэструсе, эструсе и метаэструсе и дозозависимым образом повышала уровень пролактина в плазме крови у самцов крыс [4].

Ранее исследований связи Прл-РГ и тревожности не проводилось. Для большинства изученных нейропептидов в настоящее время показано наличие не только физиологических, но и психотропных эффектов. Но если психотропные эффекты кортиколиберина, гонадолиберина, окситоцина, вазопрессина и т.д. активно исследуются, то влияние пролактолиберина на поведение и психику в настоящее время изучено слабо — Прл-РГ считается анорексигенным (снижающим потребление пищи) нейропептидом, который в основном играет роль в регуляции потребления пищи и расхода энергии [5]. Производные Прл-РГ обладают широким терапевтическим потенциалом [6], поскольку Прл-РГ проявляет не только анорексигенные, но и нейропротекторные свойства [7] и участвует в стрессорной реакции [8].

Для врачей эти данные могут представлять интерес, поскольку медицинские препараты, как правило, имеют побочные эффекты. Часто эти эффекты связаны с изменением эмоционального фона. Именно потому, что Прл-РГ и его синтетические аналоги обладают терапевтическим потенциалом, важно учитывать возможное влияние Прл-РГ на эмоциональную сферу, изучению чего и посвящена данная работа.

## ЦЕЛЬ ИССЛЕДОВАНИЯ

В данной работе мы исследовали влияние интраназального введения крысам Прл-РГ на тревожность — обязательный компонент стрессорной реакции организма и на реакцию социального предпочтения.

## МАТЕРИАЛЫ И МЕТОДЫ

Опыты проводили на 116 самцах крыс Вистар со средней массой 160 г в возрасте 2 мес на момент начала эксперимента в трех независимых сериях. Животных содержали в стандартных условиях при свободном доступе к пище (сухой комбикорм для грызунов) и воде, по 5 особей в клетке. Перед началом эксперимента животных в течение 14 дней ежедневно по 5 мин подвергали процедуре хэндлинга для предотвращения стрессорной реакции на взятие в руки в период проведения экспериментов [9].

Для оценки тревожности животных тестировали во всех сериях экспериментов в приподнятом крестообразном лабиринте (ПКЛ). Тревожность, определяемая по данной методике, отражает естественный страх высоты и открытых пространств у грызунов. Уровень тревожности определяется обратно пропорционально времени, проводимому в открытых рукавах.

Время тестирования составляло 5 мин. После тестирования каждой крысы лабиринт протирали раствором перекиси водорода для уничтожения запаха предыдущего животного. Тестирование проводили с 13 до 17 ч.

На первом этапе эксперимента в трех сериях изучали влияние Прл-РГ на уровень тревожности белых крыс в тесте ПКЛ с использованием различных концентраций.

Животным группы контроля (n=42) за 15 мин до теста ПКЛ вводили физиологический раствор (0,9% NaCl) по 10 мкл в каждую ноздрю. Животным из опытной группы интраназально вводили Прл-РГ (Prolactin-releasing Peptide, Hybio Pharmaceuticals) объемом по 10 мкл в каждую ноздрю (20 мкл на крысу) в дозе 10–10 моль/л (n=29) и в дозе 10–11 моль/л (n=14) за 15 мин до теста.

Во второй серии экспериментов (n=31), через неделю после тестирования в ПКЛ, поведение животных исследовали в тесте «социальное предпочтение».

На ограниченном стенками (высота 35 см) квадратном поле (90×90×70 см) в противоположных углах размещали маленькие клетки, в которых помещали крыс. Клетки были изготовлены из пластмассовых решеток (18×18×18 см), что обеспечивало в ходе эксперимента визуальный, обонятельный и тактильный контакты между животными, но предотвращало агонистические взаимодействия. Подробнее методика описана ранее (Виноградова и др., 2009) [9].

Исследуемых животных делили на две группы. Первой группе (опыт) вводили Прл-РГ по 10 мкл в концентрации 10-10М в каждую ноздрю (20 мкл на крысу) за 15 мин до теста. Второй группе, контрольным животным, вводили 0,9% NaCl (20 мкл на крысу). В одну клетку помещали крысу-чужака — животное, которое ранее ни разу не имело никаких социальных контактов с исследуемым животным, в другую — крысу из той же жилой клетки, что и тестируемое животное. Исследование проводили в светлое время суток с 13 до 16 ч, длительность теста — 5 мин. Тестируемое животное помещали в центр поля. Учитывалось время нахождения крысы на площади рядом с каждой из клеток (10% от общей свободной площади) и на нейтральном пространстве (80%). Все эксперименты снимались на видеокамеру.

Видеозапись поведения животных проводили на вебкамеру Logitech Webcam (Switzerland).

Статистическая обработка проводилась с использованием непараметрических методов статистики: критерия U Манна–Уитни в программе SPSS-17. За уровень статистической значимости принято значение p<0,05.

## ЭТИЧЕСКАЯ ЭКСПЕРТИЗА

Исследование было проведено в соответствии с правилами, принятыми Европейской конвенцией по защите позвоночных животных (Страсбург, 1986) [10]; Приказом МЗ РФ №119 Н от 1 апреля 2016 г. «Об утверждении Правил лабораторной практики», Principles of Good Laboratory Practice (OECD, ENV/MC/CUEM (98) 17, 1997); ГОСТ 33044-2014 «Принципы надлежащей лабораторной практики» (идентичен GLP OECD); со статьей 11 Федерального закона от 12 апреля 2010 г. №61-ФЗ «Об обращении лекарственных средств» (ред. от 22.10.14). Были предприняты надлежащие меры по соблюдению биоэтических норм сокращения количества исследуемых животных. На проведение экспериментов было получено разрешение этического комитета в области исследований на животных Санкт-Петербургского государственного университета.

## РЕЗУЛЬТАТЫ

Введение Прл-РГ в концентрации 10-10 ммоль приводило к тому, что крысы проводили меньше времени в открытых рукавах лабиринта, демонстрировали меньшее количество свешиваний и заходов в открытые рукава, чем животные контрольной группы, что свидетельствует о более высоком уровне тревожности у них (табл. 1). Достоверных различий по двигательной активности, количеству стоек, частоте и длительности груминга между отдельными группами не выявлено. Введение Прл-РГ в концентрации 10-11 ммоль не оказывало влияния ни на один из оцениваемых параметров поведения в ПКЛ.

В тесте социального предпочтения средние значения времени, проведенного со «своим» или «чужим» у животных, которым вводили Прл-РГ, достоверно не отличались от данных, полученных в группе контроля (табл. 2). Однако оказалось, что эффект Прл-РГ зависит от исходного уровня тревожности, который был определен в первой серии экспериментов (см. табл. 1). Если выделить две группы животных — низко- и высокотревожных, их реакция на Прл-РГ различна.

После введения Прл-РГ в концентрации 10-10 ммоль крысы с исходно высоким уровнем тревожности (среднее время пребывания в открытых рукавах ПКЛ 25±8 с) не демонстрировали достоверно значимого предпочтения к знакомому или незнакомому животному, т.е. у высокотревожных особей введение Прл-РГ не влияло на предпочтение «знакомца» или «незнакомца» (табл. 3).

Низкотревожные крысы (среднее время пребывания в открытых рукавах ПКЛ 146±21 с) проводили больше времени рядом с незнакомой особью, чем со знакомой. Эти данные совпадают с ранее полученными результатами (Виноградова, Лукина, Жуков, 2009) [9].

После введения Прл-РГ низкотревожные животные меньше времени проводили с чужаком, чем животные из контрольной группы (см. табл. 3), т.е. они демонстрировали предпочтение поведения, сходного с поведением высокотревожных животных группы «контроль».

**Table:** Таблица 1. Показатели поведения в приподнятом крестообразном лабиринте

	КонтрольNaCl (n=28)	Пролактолиберин 10-10 М n=29	КонтрольNaCl n=14	Пролактолиберин 10-11 М n=14
Время в открытых рукавах, с	48,7±7,4	28,2±6,2*	45±5,2	43±6,3#
Количество свешиваний	4,5±0,9	1,9±0,2*	5,8±1,1	6,3±1,2#
Количество стоек	6,8±0,9	6,1±1,1	7,1±1,1	7,2±1,1
Двигательная активность, см	282±21,2	253±28,3	272±22,8	261±30,2
Акты груминга	2,8±0,5	3,5±0,4	3,2±0,9	5,8±1,2

**Table:** Таблица 2. Социальное предпочтение в тесте «свой-чужой». Среднее время, проводимое крысами на разных участках экспериментальной камеры

	Время, проводимое со «своим», с	Время, проводимое с «чужим», с	Время, проводимое на нейтральной территории, с
Контроль (n=15)	77,9±20,6	143,3±29,7	78,5±11,0
Прл-РГ 10-10 М (n=16)	117±20,7	83,3±23,3	100,6±14

**Table:** Таблица 3. Социальное предпочтение низко- и высокотревожных крыс в тесте «свой-чужой». Среднее время, проводимое крысами на разных участках экспериментальной камеры.

	Высокотревожные (n=12)	Низкотревожные (n=13)
	свой	чужой	нейтр.	свой	чужой	нейтр.
Контроль	110±32	100±28,8	90±27	84±18	167±26.7#	49±7,7
Про-РГ 10-10 М	104±27	89±39	107±24	131±35	78±27,0 *	91±21

## ОБСУЖДЕНИЕ

Наиболее важным результатом данного исследования является то, что интраназальное введение Прл-РГ в наномолярной концентрации приводит к увеличению уровня тревожности, но не оказывает влияния на двигательную активность у крыс. Это указывает на анксиогенный, а не седативный эффект Прл-РГ.

Вероятно, влияние введения Прл-РГ на социальное предпочтение связано именно с изменением уровня тревоги.

В контроле животные с врожденной низкой тревожностью предпочитали общество незнакомца, а животные с врожденной высокой тревожностью, наоборот, — знакомой особи [9]. Это различие связано, вероятно, с балансом мотиваций. В новой обстановке у крысы происходит столкновение двух мотиваций: самосохранения и исследовательской. Можно предположить, что у крыс с высокой базальной тревожностью мотивация самосохранения сильнее исследовательской мотивации, поэтому они предпочитают находиться рядом с животным из своей клетки. У крыс с низким уровнем тревожности менее выражена неофобия, и они на этом фоне могут позволить себе проявить интерес к незнакомому животному, т. е. исследовательскую активность.

Центральные механизмы психотропных эффектов Прл-РГ изучены слабо, но показана возможность взаимодействия Прл-РГ со структурами ЦНС, регулирующими эмоции и стрессорный ответ [6][11]. Клеточные тела, окруженные иммунореактивной меткой, обнаружены, помимо гипоталамуса, в ядре одиночного тракта и вентромедиальном продолговатом мозге [12]. Метка в нервных проекциях присутствует в гипоталамусе, а также и в таламических ядрах, в миндалине и в конечной пластинке продолговатого мозга [12]. мРНК Прл-РГ экспрессируется в ядре одиночного тракта ствола мозга в несколько меньшей степени — в дорзомедиальном ядре гипоталамуса и в вентролатеральном ретикулярном ядре таламуса [12][13].

Рецептор, обладающий высоким сродством к Прл-РГ, назван GPR10 (связанный с G-белком 10) [2]. мРНК GPR10 экспрессируется в нескольких отделах мозга крысы, в первую очередь в таламусе, гипоталамусе и в area postrema [12][14]. Рецептор GPR10 найден и в парабрахиальном ядре, и в прилежащем ядре [1], структурах ноцицептивной системы, а также в гиппокампе [15], участвующем в реализации многочисленных функций, в том числе и стрессорного ответа организма.

Несомненно участие Прл-РГ в стрессорной реакции [8][16]. Нейроны срединного возвышения, продуцирующие Прл-РГ, активируются при электро-болевом раздражении [17]. Мыши с нокаутным геном Прл-РГ реагируют на иммобилизационный стресс иначе, чем контрольные животные: у нокаутов повышено содержание глюкозы и кортикостерона в крови [18]. Прл-РГ-ергические нейроны ядра одиночного тракта активируются стрессорными стимулами [19]. Внутрижелудочковое введение Прл-РГ увеличивает концентрацию кортикостерона и окситоцина в крови, а введение антисыворотки Прл-РГ препятствует стрессорной активации паравентрикулярного ядра гипоталамуса и уменьшает выделение окситоцина в кровь [20].

## ЗАКЛЮЧЕНИЕ

Подводя итог, наше исследование впервые показало, что Прл-РГ участвует и в эмоциональном компоненте стрессорной реакции: интраназальное введение Прл-РГ увеличивает тревожность крыс, что проявляется в избегании открытых рукавов ПКЛ и в снижении интереса к незнакомым особям в тесте социального предпочтения. Механизмы этих эффектов, а также возможная роль Прл-РГ в формировании тревожных и депрессивных расстройств требуют дальнейшего изучения, но уже сейчас очевидны возможности создания новых противотревожных препаратов на основе производных Прл-РГ.

## ДОПОЛНИТЕЛЬНАЯ ИНФОРМАЦИЯ

Источники финансирования. Плановая НИР Института физиологии им. И.П. Павлова РАН по теме 63.1.

Конфликт интересов. Авторы декларируют отсутствие явных и потенциальных конфликтов интересов, связанных с содержанием настоящей статьи.

Участие авторов. Все авторы одобрили финальную версию статьи перед публикацией, выразили согласие нести ответственность за все аспекты работы, подразумевающую надлежащее изучение и решение вопросов, связанных с точностью или добросовестностью любой части работы.

Благодарности. Мы благодарим П.В. Волчека за техническую помощь.

## References

[cit1] Welch S.K., Ohara B.F., Kilduff T.S., Heller H.C. (2002). Sequence and Tissue Distribution of a Candidate G-Coupled Receptor Cloned from Rat Hypothalamus. Biochemical and Biophysical Research Communications.

[cit2] Dodd Garron T., Luckman Simon M. (2013). Physiological Roles of GPR10 and PrRP Signaling. Frontiers in Endocrinology.

[cit3] Hinuma Shuji, Habata Yugo, Fujii Ryo, Kawamata Yuji, Hosoya Masaki, Fukusumi Shoji, Kitada Chieko, Masuo Yoshinori, Asano Tsuneo, Matsumoto Hirokazu, Sekiguchi Masahiro, Kurokawa Tsutomu, Nishimura Osamu, Onda Haruo, Fujino Masahiko (2002). A prolactin-releasing peptide in the brain. Nature.

[cit4] Matsumoto Hirokazu, Noguchi Jiro, Horikoshi Yasuko, Kawamata Yuji, Kitada Chieko, Hinuma Shuji, Onda Haruo, Nishimura Osamu, Fujino Masahiko (2002). Stimulation of Prolactin Release by Prolactin-Releasing Peptide in Rats. Biochemical and Biophysical Research Communications.

[cit5] Takayanagi Yuki, Matsumoto Hirokazu, Nakata Masanori, Mera Takashi, Fukusumi Shoji, Hinuma Shuji, Ueta Yoichi, Yada Toshihiko, Leng Gareth, Onaka Tatsushi (2008). Endogenous prolactin-releasing peptide regulates food intake in rodents. Journal of Clinical Investigation.

[cit6] Pražienková, Popelová, Kuneš, Maletínská (2019). Prolactin-Releasing Peptide: Physiological and Pharmacological Properties. International Journal of Molecular Sciences.

[cit7] Holubová Martina, Hrubá Lucie, Popelová Andrea, Bencze Michal, Pražienková Veronika, Gengler Simon, Kratochvílová Helena, Haluzík Martin, Železná Blanka, Kuneš Jaroslav, Hölscher Christian, Maletínská Lenka (2018). Liraglutide and a lipidized analog of prolactin-releasing peptide show neuroprotective effects in a mouse model of β-amyloid pathology. Neuropharmacology.

[cit8] Maniscalco JW, Rinaman L. Interoceptive modulation of neuroendocrine, emotional, and hypophagic responses to stress. Physiol Behav. 2017;176:195-206. doi: https://doi.org/10.1016/j.physbeh.2017.01.027 PMC5433881 28095318

[cit9] Vinogradova E.P., Zhukov D.A., Lukina E.A. Sotsial'nye predpochteniya u belykh krys s vysokim i nizkim urovnem trevozhnosti // Zhurnal vysshei nervnoi deyatel'nosti. — 2010. — T. 60. — №1. — S. 39-45.

[cit10] European Convention for the Protection of Vertebrate Animals Used for Experimental and other Scientific Purposes. European Trety Series. 1986;123:1-11. Available from: https://rm.coe.int/168007a67b

[cit11] Norris David O., Carr James A. (2020). Organization of the mammalian hypothalamus-pituitary axes. Vertebrate Endocrinology.

[cit12] Roland Barbara L., Sutton Steven W., Wilson Sandy J., Luo Lin, Pyati Jayashree, Huvar Rene, Erlander Mark G., Lovenberg Timothy W. (2014). Anatomical Distribution of Prolactin-Releasing Peptide and Its Receptor Suggests Additional Functions in the Central Nervous System and Periphery. Endocrinology.

[cit13] Morales T., Hinuma S., Sawchenko P. E. (2003). Prolactin-Releasing Peptide is Expressed in Afferents to the Endocrine Hypothalamus, but not in Neurosecretory Neurones. Journal of Neuroendocrinology.

[cit14] Ibata Yasuhiko, Iijima Norio, Kataoka Yuko, Kakihara Kenshi, Tanaka Masaki, Hosoya Masaki, Hinuma Shuji (2002). Morphological survey of Prolactin-releasing peptide and its receptor with special reference to their functional roles in the brain. Neuroscience Research.

[cit15] Špolcová Andrea, Mikulášková Barbora, Holubová Martina, Nagelová Veronika, Pirnik Zdenko, Zemenová Jana, Haluzík Martin, Železná Blanka, Galas Marie-Christine, Maletínská Lenka (2018). Anorexigenic Lipopeptides Ameliorate Central Insulin Signaling and Attenuate Tau Phosphorylation in Hippocampi of Mice with Monosodium Glutamate-Induced Obesity. Journal of Alzheimer's Disease.

[cit16] Maniscalco JW, Rinaman L. Interoceptive modulation of neuroendocrine, emotional, and hypophagic responses to stress. Physiol Behav. 2017;176:195-206. doi: https://doi.org/10.1016/j.physbeh.2017.01.027 PMC5433881 28095318

[cit17] Morales T, Sawchenko P.E (2003). Brainstem prolactin-releasing peptide neurons are sensitive to stress and lactation. Neuroscience.

[cit18] Mochiduki A., Takeda T., Kaga S., Inoue K. (2010). Stress Response of Prolactin-Releasing Peptide Knockout Mice as to Glucocorticoid Secretion. Journal of Neuroendocrinology.

[cit19] Card J. Patrick, Johnson Aaron L., Llewellyn-Smith Ida J., Zheng Huiyuan, Anand Rishi, Brierley Daniel I., Trapp Stefan, Rinaman Linda (2018). GLP-1 neurons form a local synaptic circuit within the rodent nucleus of the solitary tract. Journal of Comparative Neurology.

[cit20] Zhu L.L, Onaka T (2003). Facilitative role of prolactin-releasing peptide neurons in oxytocin cell activation after conditioned-fear stimuli. Neuroscience.

